# Genetically Engineered Macrophages Derived from iPSCs for Self-Regulating Delivery of Anti-Inflammatory Biologic Drugs

**DOI:** 10.1155/2024/6201728

**Published:** 2024-01-06

**Authors:** Molly Klimak, Farshid Guilak

**Affiliations:** ^1^Department of Orthopaedic Surgery, Washington University, St. Louis, MO 63110, USA; ^2^Shriners Hospitals for Children–St. Louis, St. Louis, MO 63110, USA; ^3^Department of Biomedical Engineering, Washington University, St. Louis, MO 63110, USA; ^4^Center of Regenerative Medicine, Washington University, St. Louis, MO 63110, USA

## Abstract

In rheumatoid arthritis, dysregulated cytokine signaling has been implicated as a primary factor in chronic inflammation. Many antirheumatic and biological therapies are used to suppress joint inflammation, but despite these advances, effectiveness is not universal, and delivery is often at high doses, which can predispose patients to significant off-target effects. During chronic inflammation, the inappropriate regulation of signaling factors by macrophages accelerates the progression of disease by driving an imbalance of inflammatory cytokines, making macrophages an ideal cellular target. To develop a macrophage-based therapy to treat chronic inflammation, we engineered a novel induced pluripotent stem cell (iPSC)-derived macrophage capable of delivering soluble TNF receptor 1 (sTNFR1), an anti-inflammatory biologic inhibitor of tumor necrosis factor alpha (TNF-*α*), in an autoregulated manner in response to TNF-*α*. Murine iPSCs were differentiated into macrophages (iMACs) over a 17-day optimized protocol with continued successful differentiation confirmed at key timepoints. Varying inflammatory and immunomodulatory stimuli demonstrated traditional macrophage function and phenotypes. In response to TNF-*α*, therapeutic iMACs produced high levels of sTNFR1 in an autoregulated manner, which inhibited inflammatory signaling. This self-regulating iMAC system demonstrated the potential for macrophage-based drug delivery as a novel therapeutic approach for a variety of chronic inflammatory diseases.

## 1. Introduction

Rheumatoid arthritis (RA) is a painful and debilitating joint disease affecting 1% of the population worldwide [1]. The pathogenesis of RA is believed to be mediated by increased levels of proinflammatory cytokines, such as interleukin (IL)-1*α*/*β* and tumor necrosis factor alpha (TNF-*α*), which drive chronic inflammation, cartilage destruction, and pain [2–5]. Advances in disease-modifying antirheumatic drugs (DMARDs) have led to the development of therapies that can reduce joint inflammation and pain, two of the leading causes of work disability in RA patients. However, while partially effective, these treatments are often delivered at high, immunosuppressive doses, which can predispose patients to significant and debilitating off-target effects while also failing to fully mitigate disease [6, 7]. Furthermore, current treatments do not provide the temporal specificity and precise control necessary to treat fluctuating RA symptoms. In this regard, a self-regulating cell-based therapy could provide a sensitive, safe, and specific system for the long-term delivery of biologic drugs in a therapeutic manner, mitigating adverse events triggered by conventional RA biologics [8].

A candidate cell type for cell-based biologic drug delivery is the macrophage, a key immune cell highly prevalent in RA that shows capabilities for homing and engraftment to sites of inflammation [9]. During chronic inflammation, the dysregulated signaling by macrophages drives an imbalance of inflammatory cytokines, accelerating the progression of disease [2, 10–13]. Initial inflammatory signaling by macrophages perpetuates the inflammatory environment of RA through numerous downstream mediators including the recruitment of additional immune cells, induction of T-cell differentiation, and increased bone resorption processes [11]. Thus, targeting downstream byproducts of this inflammation can limit harmful side effects. However, the self-propagation (e.g., positive feedback loop) of inflammatory signaling by macrophages makes attenuation of chronic inflammation challenging. Furthermore, modifying cells of the innate immune system is an ongoing challenge, and novel approaches are necessary to harness macrophage signaling for RA treatment [14]. Here, we propose the use of “smart” macrophages that possess the capacity to deliver precisely controlled anticytokine drugs in a self-regulating manner.

**Figure 1:**
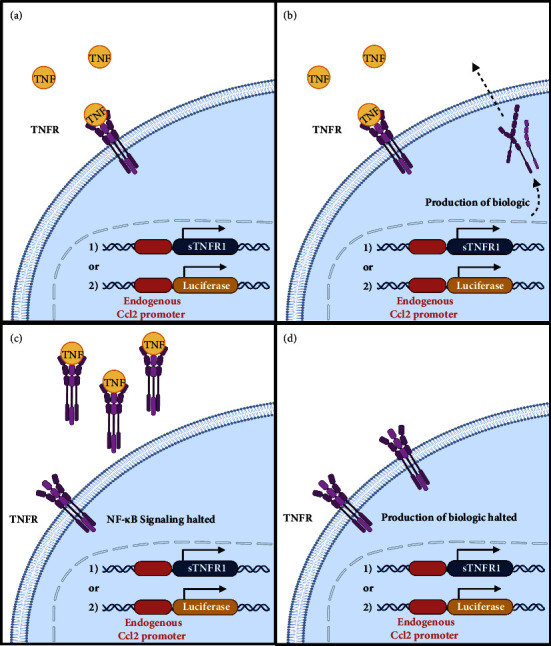
Depiction of the reprogrammed inflammatory signaling pathway in CRISPR-Cas9-engineered iMACs for the biologic Ccl2-sTNFR1 (1) and reporter Ccl2-luciferase (2) circuits. (a) TNF signaling through the TNFR type 1 receptor initiates a cascade leading to nuclear translocation and increased transcriptional activity of NF-*κ*B, activating an inflammatory transcriptional program. (b) The Ccl2 promotor is then activated, which induces the expression of soluble TNF type 1 receptor (sTNFR1) in the biologic circuit (1) and luciferase in the reporter circuit (2). (c) Upon antagonism of TNF in the microenvironment, signal transduction through TNFR1 and the NF-*κ*B inflammatory cascade is inhibited. (d) Expression of the sTNFR1 transgene decreases upon inhibition of NF-*κ*B initiating inflammation.

The use of cell therapies based on pluripotent stem cells has gained significant interest in the field of immunoengineering [15–18]. Recent advances in the fields of synthetic biology and genome editing have enabled rapid and precise engineering of the cellular genome, allowing researchers to immunomodulate previously difficult targets for a wide array of applications [19–23]. CRISPR-Cas9 genome editing was used to reprogram murine-induced pluripotent stem cells (miPSCs) as a basis for developing cell-based biologic delivery. Specifically, monocyte chemoattractant protein-1 (MCP-1, gene name *Ccl2*), a chemotaxis-inducing molecule produced downstream of TNF-*α* or IL-1 signaling, was targeted to create genetic circuits with a gene addition on one allele, as not to compromise overall cellular function, and to generate miPSCs encoding: (1) Ccl2-luciferase (Luc)—a firefly luciferase transcriptional reporter, or (2) Ccl2-sTNFR1—chimeric human sTNFR1-murine immunoglobulin G (Figure 1(a)) [15]. In these circuits, TNF-*α* signaling leads to the activation of NF-*κ*B inflammatory cascade and the induction of *Ccl2* (Figure 1(b)). Subsequent release of sTNFR1 under the Ccl2 locus (Figure 1(c)) results in the inhibition of TNF-*α* signaling, ceasing induction of *Ccl2* expression and sTNFR1 production (Figure 1(d)). Thus, these two genetic circuits act to confer cytokine-activated and feedback-controlled gene expressions, with the Ccl2-Luc line driving luciferase expression to allow imaging for circuit activation. In this study, we demonstrated the successful differentiation and development of a novel iPSC-derived engineered macrophage (iMAC) capable of responding to inflammation and delivering precisely controlled anticytokine drugs in a self-regulating manner. These engineered macrophages provide an effective proof of concept for an innovative framework extending previous systems of immunoengineered drug delivery vehicles for anti-inflammatory applications.

## 2. Methods

### 2.1. Cell Culture and Differentiation

**Figure 2:**
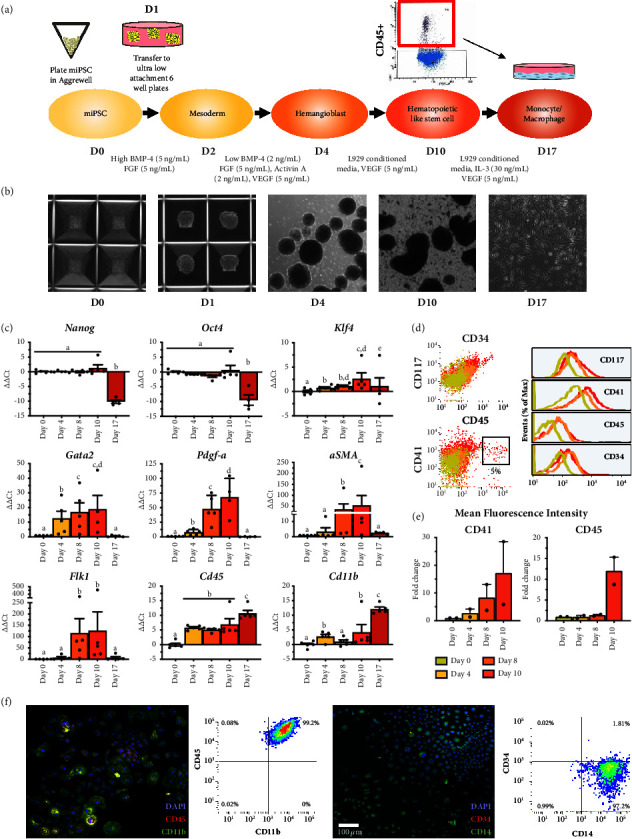
(a) Overview of the differentiation protocol for iMAC differentiation from miPSC using stepwise daily feeding with various growth factors and media conditions. (b) Representative images at each stage of differentiation throughout the protocol. (c) qPCR throughout differentiation, normalized to reference gene GAPDH expression at day 0; different letters denote *p* < 0.05 by one-way ANOVA over time in 4-5 independent experiments. (d) Representative flow cytometry of two independent experiments for both early and late markers of primitive hematopoiesis and (e) quantified for mean fluorescence intensity, demonstrated varying but similar increase in marker expression. (f) Representative images of immunocytochemistry of day 17 iMACs for macrophage markers CD11b/CD45 and CD14/CD34 (*n* = 6) and quantified by flow cytometry (*n* = 2). Bars represent mean ± SEM.

Three murine iPSC lines, one unedited and two edited lines, that were previously generated from tail fibroblasts from adult C57BL/6 mice and validated for pluripotency, were maintained on mitomycin C-treated mouse embryonic fibroblasts (MEFs) (EMD Millipore™ EmbryoMax™ PMEFNL, Fisher Scientific) [24, 25]. Unedited miPSCs were compared to an unedited miPSC line purchased from the Gates Center for Regenerative Medicine (University of Colorado, Denver) throughout differentiation to validate reproducibility between lines [26]. To generate embryoid bodies (EBs), MEFs were feeder subtracted and miPSCs from all lines were seeded at 3 × 10^6^ cells/well in an 800 *μ*m 24-well AggreWell plate (STEMCELL Technologies) coated with antiadherence rinsing solution (STEMCELL Technologies) to decrease cell attachment. Cells were cultured for 24–48 hours in the microwell plates until spheres formed. Spheres were detached from AggreWell plates and cultured on ultralow attachment 6-well plates at ∼300 spheres/well. EBs were digested into single-cell suspensions using 12.5 mg/mL collagenase II (Sigma-Aldrich) and 660 PKU/ml pronase (EMD Millipore) after hematopoietic differentiation and sorted by flow cytometry for CD45^+^ cells. Single cells were then seeded onto 48-well plates at 15,000 cells/well and cultured for an additional week (Figure 2(a)). Growth factors used throughout differentiation include iPSC stage—Recombinant Mouse LIF protein (R and D Systems, 8878-LF); day 0–10—Mouse BMP-4 Recombinant protein (Fisher Scientific, 5020BP010), Recombinant Mouse FGF basic protein (Fisher Scientific, 3139FB025), Recombinant Human/Mouse/Rat Activin A protein (Fisher Scientific, 338AC050), and Human VEGF 165 Recombinant protein (Fisher Scientific, 293-VE-010); day 10–17—Recombinant Murine IL-3 (PeproTech, 213-13).

L929 conditioned media was generated by culturing L929 cells for 7 days in DMEM/F12, 10% FBS, and 1% penicillin/streptomycin. Media were collected, filtered, and stored at −20°C until use. Bone-marrow-derived macrophages were generated by first isolating the bone marrow from long bones in C57BL/6 mice. Bone marrow was then incubated with red cell lysis buffer and strained with a 40 *μ*m strainer. Isolated cells were cultured for 7–10 days in DMEM-HG with 10% FBS, 1% penicillin/streptomycin, and 30% L929 conditioned media prior to testing.

### 2.2. Flow Cytometry

Cells were passed through a 40 *μ*m strainer to remove debris and blocked with Fc receptor antibody CD16/32 to prevent nonspecific antibody binding before staining. Dead cells were stained with propidium iodide and examined for differentiation markers (Supplementary Table 1) in a cell staining buffer (Biolegend). Doublets and cellular debris were excluded through FlowJo analysis. For cell sorting, similar methods were utilized to stain CD45-APC positive cells, which were sorted using a FACS Aria II (BD Bioscience).

### 2.3. RNA Isolation

Following experimental treatment, culture media were collected, and cells were imaged and then rinsed in PBS, lysed in RL buffer, snap frozen, and stored at −80°C until RNA isolation. RNA was isolated according to the manufacturer's recommendations (Total RNA Purification Plus Kit; Norgen Biotek).

### 2.4. Gene Expression with RT-qPCR

200 ng of RNA was reverse transcribed using SuperScript VILO complementary DNA master mix (Invitrogen). Real-time PCR was performed using Fast SyBR Green Master Mix (Applied Biosystems) on a QuantStudio (ThermoFisher) with 10 ng of cDNA and primer concentration at 10 *μ*M (Supplementary Table 2). All primers were validated prior to use between 90 and 110% efficiency. All reactions were performed in duplicate for each analyzed gene. Differences in gene expression were calculated as fold change using the ΔΔCT method: fold change was normalized to no treatment or day 0 with GAPDH as the housekeeping gene where GAPDH maintained consistent expression throughout all differentiation timepoints and experimental treatments.

### 2.5. Western Blot

Cells were rinsed with PBS, incubated on ice in RIPA buffer for 5 minutes, collected, and centrifuged at 14,000 × *g* for 10 minutes to pellet the cellular components. Proteins were denatured in a Laemmli sample buffer (Bio-Rad) at 95°C for 5 minutes, separated by standard SDS/PAGE using 7.5% polyacrylamide gels, and transferred to a PVDF membrane. Membranes were rinsed with PBS/Tween20 buffer and blocked at room temperature for 1 hour with 5% milk in PBS/Tween20. After 1 hour of washing, membranes were probed with primary antibodies 1 : 3000 sheep anti-Arginase 1 (Fisher Scientific, AF5868), 1 : 200 mouse anti-CD206 (R&D Systems, AF2535-SP), 1 : 2000 rabbit anti-iNOS (Cell Signaling Technologies 2982S), 1 : 1000 rabbit anti-GAPDH (Proteintech, 10494-1-AP), and 1 : 2000 goat anti-Cyclophilin A (Fisher Scientific, AF3589SP) at 4°C overnight, rinsed, and then incubated with secondary antibodies at 1 : 1000 donkey antisheep IgG (Abcam, ab6900), 1 : 1000 goat antirabbit IgG (Cell Signaling Technologies, 7074P2), 1 : 1000 donkey antigoat (Abcam, ab97110), and 1 : 2000 rabbit antimouse IgG (Abcam, ab97046) at room temperature for 1 hour. Blots were developed with Pierce ECL Western Blotting Substrate and imaged with a ChemiDoc XRS.

### 2.6. Boyden Cell Migration Assay

Cells were prepared by trypsinization and incubated in starvation media (media with 5% FBS) for 1 hr. Cells were resuspended in DMEM at a concentration of 5 × 10^5^ cells/mL. Polycarbonate PFB filters (Neuro Probe) with 8 *μ*m pores were used. Culture media with 20 ng/ml of TNF-*α* or 100 ng/ml MCP1 were placed in the bottom chamber of Neuro Probe 48-well chemotaxis chambers (Neuro Probe) with 5 × 10^4^ cells in the top chamber and incubated at 37°C in 95% O_2_/5% CO_2_ for 24 h. Migrated cells were fixed in methanol and stained with 500 nM DAPI.

### 2.7. Immunocytochemistry

Cells were fixed in 2% paraformaldehyde for 30 minutes, washed, and then blocked at ambient in 5% normal goat serum for one hour. Cells were then stained with CD45-APC, CD34-PE, CD14-FITC, or CD11b-Alexa 488 (1 : 500) for one hour at ambient before being washed and then stained with DAPI for five minutes at ambient (1 : 4000). Images were captured using a confocal laser scanning microscope (LSM 880, Zeiss).

### 2.8. Additional Assays

Luciferase activity from Ccl2-Luc cells in all monolayer experiments was measured using the Bright-Glo luminescence assay (Promega) and a Cytation5 plate reader. Luciferase activity is reported as relative luminescence.

Culture media were collected from samples and stored at −20°C. sTNFR1 concentration was measured with DuoSet enzyme-linked immunosorbent assay specific to human sTNFR1 (R&D Systems). Each sample was measured in technical duplicates, and absorbance was measured at 450 and 540 nm.

As nitric oxide production has been well documented as a major player in inflammation, nitric oxide production in the supernatant was measured using a Griess assay. Cell supernatant was collected, and the concentration of nitric oxide within the culture medium was measured by the Griess reagent (Promega) according to the manufacturer's instructions.

A phagocytosis assay (Cayman Chemical) was performed according to the manufacturer's instructions to measure the phagocytic ability of iMACs, BMDMs, and miPSCs. Briefly, cells were treated with FITC-labeled latex beads or control media for 4 hours (1 : 200). Excess beads were removed by washing with PBS, and cells were scraped in 1 mL of assay buffer, fixed, stained with CD14 and DAPI, and quantified through flow cytometry for cellular uptake (BD X-20).

### 2.9. Statistical Analysis

Statistical analysis was performed with GraphPad Prism using analysis of variance (ANOVA) (*α* = 0.05) with Tukey's HSD post hoc test. For qRT-PCR comparisons, all data were normalized to no treatment or time zero as the control and fold-change values were log-transformed prior to statistical analysis. For all data, standard error of means was used (SEM).

## 3. Results

### 3.1. Murine iPSCs Successfully Differentiate into a Macrophage Lineage

The primary goal of this work was to investigate the potential of using CRISPR-Cas9 reprogrammed iPSCs to derive macrophages as an engineered cell therapy with the capacity to respond to an inflammatory stimulus and deliver anticytokine drugs in a precisely controlled and self-regulating manner. Therefore, it was first necessary to develop a protocol that could reliably differentiate macrophages from miPSCs. Published protocols were tested to obtain an optimized differentiation protocol for the successful development of macrophages from multiple wild type and edited miPSC lines [27–30]. Briefly, this protocol relies upon embryoid body cell culture with daily feeding of a distinct set of growth factors and small molecules (Figures 2(a) and 2(b)). To characterize the generated iMACs, successive qPCR testing for differentiation markers was completed at day 0, 4, 8, 10, and 17 over multiple differentiations in two unique cell lines (Figure 2c; Supplementary Figure 1). As a point of reference, all timepoints were compared to day 0 of differentiation. Gene expression throughout the protocol demonstrated distinct waves in expression. Early-stage pluripotency genes (*Nanog* and *Oct4*) were significantly downregulated by terminal differentiation (Figure 2c). Key hemangioblast and hematopoietic genes (*Klf4*, *Gata2*, *Pdgf-a*, *αSMA*, and *Flk1*) increased in expression during primitive hematopoiesis (day 0–10) but lost expression by the terminal macrophage endpoint while macrophage differentiation markers (*Cd45* and *Cd11b*) increased over time. Additional flow cytometry and immunocytochemistry analysis were performed examining stem cell and macrophage markers. Flow cytometry for both early (CD117) and late (CD41 and CD45) markers of primitive hematopoiesis as well as myeloid precursor (CD34) further corroborated successful differentiation (Figures 2(d) and 2(e), Supplementary Figure 1A). Immunocytochemistry on fully differentiated iMACs showed macrophage marker expression (CD11b, CD14, and CD45) throughout the entire population by day 17 in comparison to an unstained control, while earlier lineage marker CD34 was not present and was confirmed by flow cytometry where differentiated macrophages were largely CD45/CD11b/CD14^+^ and CD34^−^ (Figure 2(f); Supplementary Figure 2).

### 3.2. iMACs Respond Similarly in Phenotype and Function to Bone Marrow-Derived Macrophages

**Figure 3:**
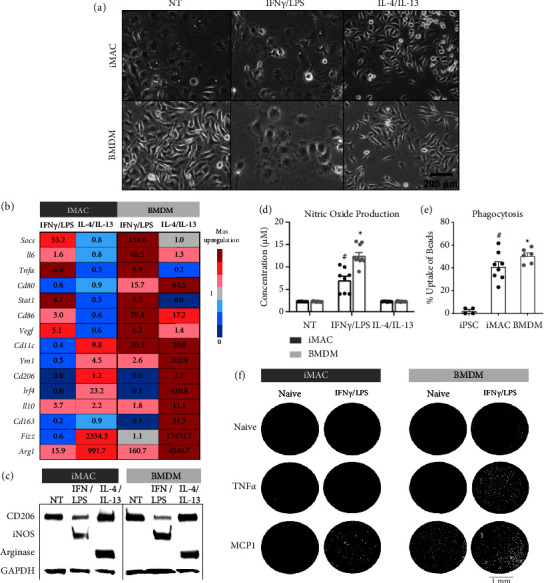
Polarization and signaling in response to cytokine treatment with either no treatment (NT), IFN*γ*/LPS, or IL-4/IL-13 stimulus after 24 hours. (a) Phase contrast imaging of iMAC in comparison to BMDMs. (b) qPCR normalized to GAPDH demonstrated similar upregulated gene expression in response to inflammatory or immunomodulatory stimuli, with lower overall fold-change response for iMACs than in BMDMs. Heat map color represents downregulation (blue), upregulation (red), or no change (grey) per gene (*n* = 6). (c) Western blot analysis of iMACs/BMDMs cytokine activation. (d) Griess assay for nitric oxide production of iMAC/BMDMs in response to inflammatory stimuli. (e) Phagocytosis in iMACs/BMDMs quantified by flow cytometry. (f) Migration images from Boyden chamber for iMACs/BMDMs, naïve, or prestimulated with IFN*γ*/LPS, in response to chemoattractants (*n* = 4). (d, e) Bars represent mean ± SEM with # (iMAC) or *∗* (BMDM) denoting *p* < 0.05 to no treatment by one-way ANOVA.

We then further characterized the phenotype of our differentiated unedited miPSC-derived macrophages as functionally differentiated macrophages as compared to the well-studied murine bone-marrow-derived macrophage (BMDM) model. Generated iMACs at day 17 and fully differentiated BMDMs were stimulated for 24 hours in either no treatment, 100 ng/mL IFN*γ* and 1 ng/mL LPS, or 10 ng/mL IL-4 and 10 ng/mL IL-13 and evaluated in terms of morphology, activation, and function. Cell morphology remained consistent between iMACs and BMDMs with treatment (Figure 3(a)). When stimulated with IFN*γ*/LPS or IL-4/IL-13 for 24 hours, qPCR gene expression analysis demonstrated that iMACs activated similarly to BMDMs, significantly increasing expression (*p* < 0.05, *n* = 4) of inflammatory genes (*Socs*, *Il6*, *Tnfa*, *Cd80*, *Stat1*, *Cd86*, and *Vegf*) in response to IFN*γ*/LPS and immunomodulatory genes (*Cd11c*, *Ym1*, *Cd206*, *Irf4*, *Il10*, *Cd163*, *Fizz*, and *Arg1*) in response to IL-4/IL-13, in comparison to a nontreated control (Figure 3(b), Supplementary Figure 3). Interestingly, though similar in pattern, iMACs exhibited a dampened response to cytokine stimulus as compared to BMDMs, demonstrating an overall lower fold change in response to all cytokine treatments than BMDMs. Western blot analysis of NOS2, CD206, and arginase with a cyclophilin A/GAPDH loading control indicated similar regulation in protein expression by both iMACs and BMDMs with increased expression of NOS2 and arginase in response to IFN*γ*/LPS and IL-4/IL-13 stimuli, respectively, with no response without treatment (Figure 3(c)). CD206 was expressed in all cells at a basal level but decreased in response to an inflammatory stimulus and increased in response to an immunomodulatory stimulus.

In regard to function, both iMACs and BMDMs produced a significant amount of nitric oxide (*p* < 0.05, *n* = 3) in response to inflammatory activation, though production by BMDMs was 2-fold higher (Figure 3(d)). Following incubation with latex beads, cells at the miPSC stage did not uptake beads as expected; both iMACs and BMDMs phagocytized beads with similar efficiency of 50–60% cellular uptake after excluding CD14^−^ cells (Figure 3(e), Supplementary Figure 1B). To evaluate the ability of iMACs to home in response to both cytokine and chemokine signals, both iMACs and BMDMs were treated with either no treatment or IFN*γ*/LPS for 24 hours to represent homing in response to inflammatory signals. Following a 24-hour incubation in a Boyden chamber, BMDMs showed higher migration in response to both a cytokine (TNF-*α*) and chemokine (CCL2/MCP-1) gradient in comparison to iMACs, where low-level migration was only observed in response to CCL2/MCP-1 (Figure 3(f)).

### 3.3. Therapeutic iMAC Mitigated Inflammatory Signaling by the Self-Regulatory Production of sTNFR1

**Figure 4:**
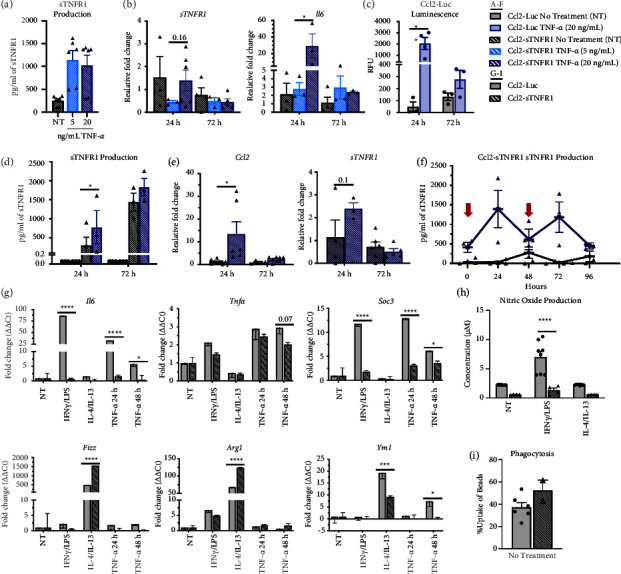
(a) ELISA for sTNFR1 in response to varying levels of TNF in Ccl2-sTNFR1 iMACs 72 hours following treatment (*n* = 3–6). (b) qPCR normalized to GAPDH in response to treatment with TNF-*α* after 24 and 72 hours in sTNFR1 edited iMACs (*n* = 3–5). (c) Ccl2-Luc iMAC luciferin production in response to TNF-*α* compared to no treatment (*n* = 3). (d) ELISA for sTNFR1 in response to TNF-*α* in Luc/sTNFR1 iMACs (*n* = 3-4). (e) qPCR normalized to GAPDH in response to treatment with TNF-*α* after 24 and 72 hours in sTNFR1 edited iMACs (*n* = 3–5). (f) ELISA over time for sTNFR1 iMACs in response to repeated stimulations as shown by red arrows (media change with either no treatment or 20 ng/mL TNF-*α*) in sTNFR1 iMACs (*n* = 3). (g) qPCR normalized to GAPDH and an untreated control in response to treatment with IFN*γ*/LPS or IL-4/IL-13 after 24 hours or to TNF-*α* after 24 and 48 hours (*n* = 3). (h) Griess assay for nitric oxide production in response to inflammatory stimuli (*n* = 4–8). (i) Phagocytosis in nontreated Ccl2-Luc and Ccl2-sTNFR1 iMACs quantified by flow cytometry, and technical replicates represent 50,000 events. Bars represent mean ± SEM, ^*∗*^*p* < 0.05, ^*∗∗*^*p* < 0.01, ^*∗∗∗*^*p* < 0.001, and ^*∗∗∗∗*^*p* < 0.0001 by one-way ANOVA. NT: no treatment.

First, we evaluated whether iMACs expressing the sTNFR1 gene edit could produce a therapeutic in response to varying concentrations of an inflammatory stimulus. iMACs were treated with a low and high dose of TNF-*α* (5 and 20 ng/mL), and mRNA and culture media were collected at 24 and 72 hours (Figures 4(a) and 4(b)). *sTNFR1* and *Il6* gene expressions were evaluated by qRT-PCR. At both TNF-*α* concentrations, *sTNFR1* and *Il6* gene expressions were increased compared with cells given no treatment (Figure 4(b)). However, in the 5 ng/mL group, there was significantly less inflammatory activation observed and no change in expression between the 24- and 72-hour timepoints, while in the 20 ng/mL group, gene expression was significantly higher at the 24-hour timepoint than 72 hours, demonstrating downregulation in expression over time. To fully characterize the response of iMACs possessing either the luciferase (Ccl2-Luc) or Ccl2-sTNFR1 circuits to an inflammatory stimulus, Ccl2-Luc/sTNFR1 iMACs were treated with 0 or 20 ng/ml of TNF-*α* for 72 hours. Ccl2-Luc iMACs treated with 20 ng/ml of TNF-*α* expressed significant production of luminescence after 24 hours that resolved to baseline by 72 hours after stimulation as compared to the untreated control (Figure 4(c)). Similarly, in response to stimulation by TNF-*α* (20 ng/ml), Ccl2-sTNFR1 iMACs produced significantly more protein at 24 hours than no treatment (Figure 4(d)). As expected, since luminescence was attenuated by 72 hours in Ccl2-Luc cells, there was also no difference in sTNFR1 production between no treatment and TNF-*α* treatment in Ccl2-sTNFR1 iMACs at 72 hours. Examining *Ccl2* and *sTNFR1* expression in Ccl2-sTNFR1 iMACs at both the 24- and 72-hour timepoints, expression normalized to an untreated control and confirmed that circuit activity was halted by 72 hours, demonstrating self-regulation in these two systems (Figure 4(e)). When Ccl2-sTNFR1 iMACs were examined for the repeatability of response to multiple flares, autoregulation of the circuit was maintained, where again sTNFR1 presence peaked by 24 hours following each stimulation with TNF-*α* and returned to baseline by 48 hours (Figure 4(f)). This production of sTNFR1 was able to mitigate inflammatory activation in Ccl2-sTNFR1 iMACs as compared to control Ccl2-Luc iMACs, with peak protection against inflammatory gene activation demonstrated at 24 hours and significant downregulation still maintained by 48 hours following stimulation (Figure 4(g)). This modulation was not exclusively TNF-specific, with Ccl2-sTNFR1 iMACs also demonstrating protection in response to stimulation with IFN*γ*/LPS and further immunomodulatory upregulation in response to IL-4/IL-13 that led to the decrease of secreted nitric oxide in response to inflammatory activation (Figure 4(h)). However, the phagocytic capacity of Ccl2-sTNFR1 iMACs remained unchanged as compared to control Ccl2-Luc iMACs (Figure 4i).

## 4. Discussion

Here, we demonstrated a proof of concept for engineered iPSC-derived macrophages, iMACs, capable of sensing and dynamically responding to inflammation by producing the anti-inflammatory mediator sTNFR1 in a self-regulated manner. Our results optimized the generation of iPSC-derived macrophages, validating and extending previously published protocols. Macrophages differentiated from miPSC showed successful sequential differentiation into a hematopoietic stem cell-like and then macrophage lineage as confirmed by positive expression (or loss) of major differentiation factors characteristic of each stage of development. While iMACs responded to stimuli in a similar pattern to BMDMs, they displayed a lower response to both cytokine and chemokine activation that led to not only reduced gene activation but also reduced nitric oxide production and migration in comparison to BMDMs. Likewise, other studies have investigated the phenotypical differences between stem cell and bone-marrow-derived macrophages that suggest a similarity between tissue-resident macrophages and iPSC- and ESC-derived macrophages formed from the primitive and transient definitive wave of hematopoiesis [31–34]. Notably, as this differentiation process relies upon the selective sorting of CD45^+^ cells, scaling up into larger-scale applications would require discussion of alternate culture strategies. As such, these findings indicated a successful proof of concept for the generation of macrophages from miPSCs that resembled tissue-resident macrophages, and further modifications of such protocols are needed to optimize macrophage differentiation, polarization, and behavior.

Ccl2 has been shown to be a crucial driver of the recruitment and proliferation of immune cells in the synovium, which results in the upregulation of inflammation and cytotoxic molecules and disruption of the immune network in the joint [35, 36]. Therefore, *Ccl2* was selected as the initial driver for therapeutic delivery. In response to TNF-*α* stimulation, iMACs possessing both the Ccl2-Luc and Ccl2-sTNFR1 gene circuits responded in a self-regulatory manner and observed through the activation and subsequent deactivation of the *Ccl2* promoter that regulated luminescence and sTNFR1 protein production, respectively. Importantly, this regulation was both rapid and uniform, consistently peaking by 24 hours and resolving by 48–72 hours in response to both single and iterative stimulations. Notably, while transgene production was resolved by 48 hours, robust anti-inflammatory effects persisted well beyond circuit autoregulation, with Ccl2-sTNFR1 iMACs maintaining significant downregulation of inflammatory genes, and increased immunomodulatory genes as compared to Ccl2-Luc iMACs. While Ccl2-Luc iMACs did not exhibit any transgene expression basally, sTNFR1 protein secretion was detected in the absence of cytokine in sTNFR1 iMACs as macrophages have been known to produce low levels of immunomodulatory factors without the activation of cytokines.

The emerging field of synthetic immunology has highlighted immune cells as ideal targets for therapeutic reprogramming where these cells can be systematically engineered to detect a multitude of environmental inputs to initiate complex, nuanced, and controlled therapeutic responses [21, 22, 37]. Macrophages provide an important target for cell therapies, as research continues to highlight their role in a broad range of diseases. They are strongly regulated by local environmental cues and possess an intrinsic homing ability, allowing them to migrate in response to these cues to sites of high inflammation, like the arthritic joint [2, 13, 38]. Indeed, the therapeutic potential of macrophage-based drug delivery strategies has been encouraging where macrophages have been used to effectively deliver multiple types of cargo [39–41]. Our work extends these efforts through the generation and engineering of macrophages that are not only capable of delivering a protein drug cargo but also possess the ability to produce multiple drugs and autoregulate their production and delivery through preprogrammed synthetic gene circuits.

Overall, this study demonstrated the successful generation of engineered iMACs for the controlled, autoregulated exogenous induction of biologics. Broadly, we examined the use of engineered iMACs in response to TNF-*α*-mediated inflammation; however, other transgenes can be utilized for developing autoregulated biological systems for a wide range of other inflammatory stimuli and disease applications. Indeed, it is our hope that this approach will emphasize the strength of macrophages, in particular iMACs, as an important overlooked therapeutic target for disease therapy, and inspire future studies examining the development of more complex circuits.

## Data Availability

The data supporting the current study are available from the corresponding author upon request.

## Supplementary Materials

Supplementary Materials Supplementary Figure 1: (A) flow cytometry for both early and late markers of primitive hematopoiesis markers as well as macrophage expression demonstrates successful differentiation in a second iPSC cell line. (B) Flow cytometry of iMACs and BMDMs demonstrated phagocytosis of latex beads with similar efficiency after excluding CD14 cells. Supplementary Figure 2: Immunocytochemistry of day 17 iMACs derived from two separate miPSC lines examining macrophage markers CD11b and CD14 as well as HSC marker CD45 and myeloid marker CD34 in comparison to a primary negative examining both low and high seeding densities (*n* = 6). Supplementary Figure 3: polarization and signaling in iMACs differentiated from multiple cell lines in response to treatment with either IFN*γ*/LPS or IL-4/IL-13 stimulus after 24 hours. PCR normalized to GAPDH suggests iMACs upregulate inflammatory/immunomodulatory gene expression similarly but demonstrate key differences between lines (*n* = 3). Supplementary Figure 4: qPCR normalized to GAPDH suggests sTNFR1 iMACs upregulate gene expression to a higher degree in response to 20 ng/mL TNF compared to 5 ng/mL (*n* = 3); mean ± SEM. Supplementary Table 1: flow cytometry antibodies. Supplementary Table 2: qPCR primer sequences.
