# Supplementary material for: Genetically Engineered Macrophages Derived from iPSCs for Self-Regulating Delivery of Anti-Inflammatory Biologic Drugs
Source: J Tissue Eng Regen Med. 2024 Jan 6;2024:6201728. doi: 10.1155/2024/6201728 (PMC10990417; doi:10.1155/2024/6201728)
Supplement: Supplementary Materials — Supplementary Figure 1: (A) flow cytometry for both early and late markers of primitive hematopoiesis markers as well as macrophage expression demonstrates successful differentiation in a second iPSC cell line. (B) Flow cytometry of iMACs and BMDMs demonstrated phagocytosis of latex beads with similar efficiency after excluding CD14 cells. Supplementary Figure 2: Immunocytochemistry of day 17 iMACs derived from two separate miPSC lines examining macrophage markers CD11b and CD14 as well as HSC marker CD45 and myeloid marker CD34 in comparison to a primary negative examining both low and high seeding densities (n = 6). Supplementary Figure 3: polarization and signaling in iMACs differentiated from multiple cell lines in response to treatment with either IFNγ/LPS or IL-4/IL-13 stimulus after 24 hours. PCR normalized to GAPDH suggests iMACs upregulate inflammatory/immunomodulatory gene expression similarly but demonstrate key differences between lines (n = 3). Supplementary Figure 4: qPCR normalized to GAPDH suggests sTNFR1 iMACs upregulate gene expression to a higher degree in response to 20 ng/mL TNF compared to 5 ng/mL (n = 3); mean ± SEM. Supplementary Table 1: flow cytometry antibodies. Supplementary Table 2: qPCR primer sequences. [file 6201728.f1.zip › Supplementary Table 2.pdf]

Supplementary Table 2. qPCR primer sequences.

Gene	Forward	Reverse
**Differentiation**		
Nanog	CAC CCA CCC ATG CTA GTC TT	ACC CTC AAA CTC CTG GTC CT
Oct 4	CCA ATC AGC TTG GGC TAG AG	CTG GGA AAG GTG TCC CTG TA
Klf4	AAC ATG CCC GGA CTT ACA AA	TTC AAG GGA ATC CTG GTC TTC
Flk1	TTG GAG CAT CTC ATC TGT TAC AGC	GGC CGG CTC TTT CGC TTA CT
Gata2	ACC ACC CTT GAT GTC CAT GT	TGC ATG CAA GAG AAG TCA CC
Pdgfa	GGACTTACCCTGGAGAAGTGAGAA	ACACCAGTTTGATGGATGGGA
CD45	CCT GAG TCT GCA TCT AAA CCC C	TGC TTG GCC AGT ATT CTG CGC A
CD11b	ATG GAC GCT GAT GGC AAT ACC	TCC CCA TTC ACG TCT CCC A
F4/80	CTT TGG CTA TGG GCT TCC AGT C	GCA AGG AGG ACA GAG TTT ATC GTG
α SMA	CTG ACA GAG GCA CCA CTG AA	AGA GGC ATA GAG GGA CAG CA
Gapdh	CAT GGC CTT CCG TGT TCC TA	TGT CAT CAT ACT TGG CAG GTT TCT
R18s	CGG CTA CCA CAT CCA AGG AA	GGG CCT CGA AAG AGT CCT GT
**Polarization**		
Il6	GAG GAT ACC ACT CCC AAC AGA CC	AAG TGC ATC ATC GTT GTT CAT ACA
CD11c	CTGGATAGCCTTTCTTCTGCTG	GCACACTGTGTCCGAACTC
Il10	GCTCTTACTGACTGGCATGAG	CGCAGCTCTAGGAGCATGTG
CD163	TCCACACGTCCAGAACAGTC	CCTTGGAAACAGAGACAGGC
CD206	CAGGTGTGGGCTCAGGTAGT	TGTGGTGAGCTGAAAGGTGA
Stat1	CTGAATATTTCCCTCCTGGG	TCCCGTACAGATGTCCATGAT
Vegf	CCTTCGTCCTCTCCTTACCC	AAGCCACTCACACACACAGC
CD80	GGCAAGGCAGCAATACCTTA	CTCTTTGTGCTGCTGATTCG
CD86	TCTCCACGGAAACAGCATCT	CTTACGGAAGCACCCATGAT
Irf4	GCAGCTCACTTTGGATGACA	CCAAACGTCACAGGACATTG
Tnfα	GGT GCC TAT GTC TCA GCC TCT T	GCC ATA GAA CTG ATG AGA GGG AG
