# Supplementary figures and images for: Genetically Engineered Macrophages Derived from iPSCs for Self-Regulating Delivery of Anti-Inflammatory Biologic Drugs
Source: J Tissue Eng Regen Med. 2024 Jan 6;2024:6201728. doi: 10.1155/2024/6201728 (PMC10990417; doi:10.1155/2024/6201728)

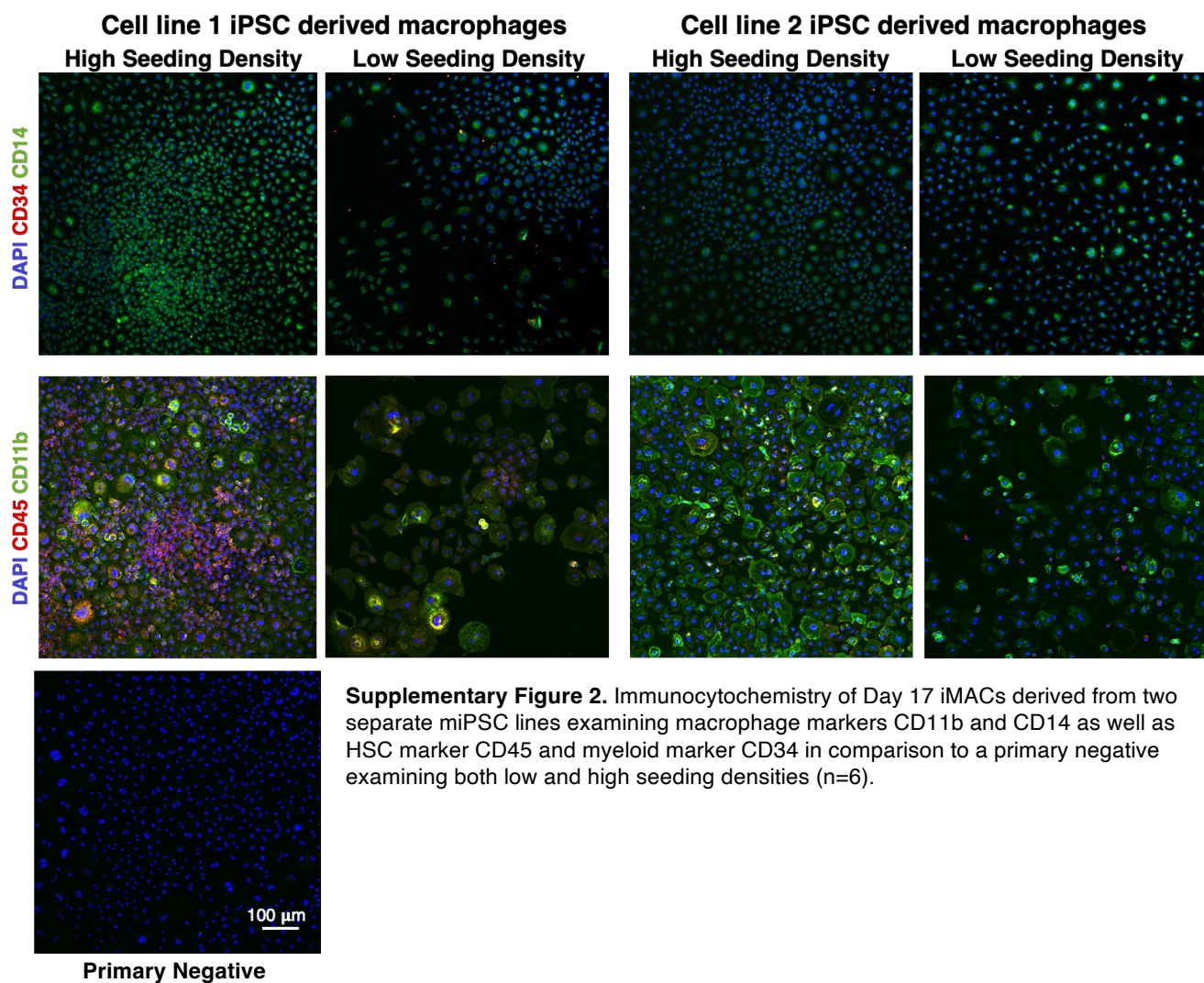

Supplement: Supplementary Materials — Supplementary Figure 1: (A) flow cytometry for both early and late markers of primitive hematopoiesis markers as well as macrophage expression demonstrates successful differentiation in a second iPSC cell line. (B) Flow cytometry of iMACs and BMDMs demonstrated phagocytosis of latex beads with similar efficiency after excluding CD14 cells. Supplementary Figure 2: Immunocytochemistry of day 17 iMACs derived from two separate miPSC lines examining macrophage markers CD11b and CD14 as well as HSC marker CD45 and myeloid marker CD34 in comparison to a primary negative examining both low and high seeding densities (n = 6). Supplementary Figure 3: polarization and signaling in iMACs differentiated from multiple cell lines in response to treatment with either IFNγ/LPS or IL-4/IL-13 stimulus after 24 hours. PCR normalized to GAPDH suggests iMACs upregulate inflammatory/immunomodulatory gene expression similarly but demonstrate key differences between lines (n = 3). Supplementary Figure 4: qPCR normalized to GAPDH suggests sTNFR1 iMACs upregulate gene expression to a higher degree in response to 20 ng/mL TNF compared to 5 ng/mL (n = 3); mean ± SEM. Supplementary Table 1: flow cytometry antibodies. Supplementary Table 2: qPCR primer sequences. [file 6201728.f1.zip › Supplemental Figure 2.pdf]
